# Congenital cysts of the lower male genitourinary tract: a disorder with various treatment approaches and pitfalls—case report

**DOI:** 10.1186/s12894-022-01048-x

**Published:** 2022-09-03

**Authors:** Matteo Moretti, Davide Campobasso, Raffaele Inzillo, Marco Grande, Francesco Facchini, Michelangelo Larosa, Jean Emmanuel Kwe, Gabriele Carlinfante, Gian Luigi Pozzoli, Maurizio Zizzo, Salvatore Micali, Antonio Frattini

**Affiliations:** 1 Urology Unit - Civil Hospital of Guastalla, AUSL-IRCCS of Reggio Emilia, Via Donatori di Sangue 1, 42016 Guastalla, RE Italy; 2 Residency Programme, University of Modena and Reggio Emilia, Modena, MO Italy; 3 Department of Pathology, Arcispedale Santa Maria Nuova, Azienda USL-IRCCS Di Reggio Emilia, Reggio Emilia, RE Italy; 4 Surgical Oncology Unit, AUSL-IRCCS of Reggio Emilia, Reggio Emilia, RE Italy; 5 Clinical and Experimental Medicine PhD Program, University of Modena and Reggio Emilia, Modena, MO Italy; 6 Urology Unit–Baggiovara Hospital, University of Modena and Reggio Emilia, Modena, MO Italy

**Keywords:** Cyst of the ejaculatory system, Malformation, Symptoms, Surgical therapy, Case report

## Abstract

**Background:**

The cysts of the male pelvic floor represent a rare clinical entity. Their origin is linked to an altered development of paramesonephric and mesonephric ducts during embryogenesis**.**

**Case presentation:**

We report our experience regarding two patients presenting cysts of the ejaculatory system treated with open and mini-invasive surgery. The patients referred to our clinic with nonspecific symptoms and the diagnosis was obtained by radiological investigations. The patient treated with an open approach developed a pelvic purulent collection and a fistula of the prostatic urethra, managed with surgical drainage and prolonged bladder catheterization. On the other hand, the patient treated with laparoscopic approach did not develop any complications. No sexual or ejaculatory disorders were reported.

**Conclusions:**

Patients with congenital cysts of the pelvic floor must be adequately informed about the risks and benefits of surgery and a careful counseling is mandatory before surgery. Treatment is recommended for symptomatic patients and an endoscopic approach is associated with a high rate of recurrence. A laparoscopic approach, when possible, is desirable.

## Background

Cysts of the male pelvic floor are uncommon and usually benign. These clinical findings are often incidental and may become symptomatic during the third and fourth decades of life [1]. Symptoms are nonspecific and may include abdominal, perineal and pelvic pain, painful ejaculation, hematospermia, lower urinary tract symptoms (LUTS), urinary retention with subsequent appearance of recurrent prostatitis, epididymitis and infertility. Moreover, these cysts have different anatomic origins and may be associated with various genitourinary congenital anomalies including hypospadias, intergender disorder, adult polycystic kidney, cryptorchidism and omolateral renal agenesis [1]. There are two principal groups of male lower urinary tract cysts: intraprostatic and extraprostatic. Intraprostatic cysts are divided in medial cysts, that develop from the prostatic utricle and Mullerian ducts, paramedial cysts (ejaculatory ducts cysts) and lateral cysts, related to prostatic retention, associated to tumors or prostatic abscesses. The extraprostatic ones include seminal vesicles, deferent ducts and Cowper ducts cysts. The differential diagnosis is usually with ureterocele, bladder diverticulum, hydroureter and ectopic ureter [2].

In this article we describe the etiopathology, diagnosis and treatment of a case study of congenital cysts of the lower male genitourinary tract.

We report our retrospective experience concerning two patients affected by extraprostatic genito-urinary cysts treated with open and mini-invasive surgery.

## Case presentation

### Case 1

**Fig. 1:**
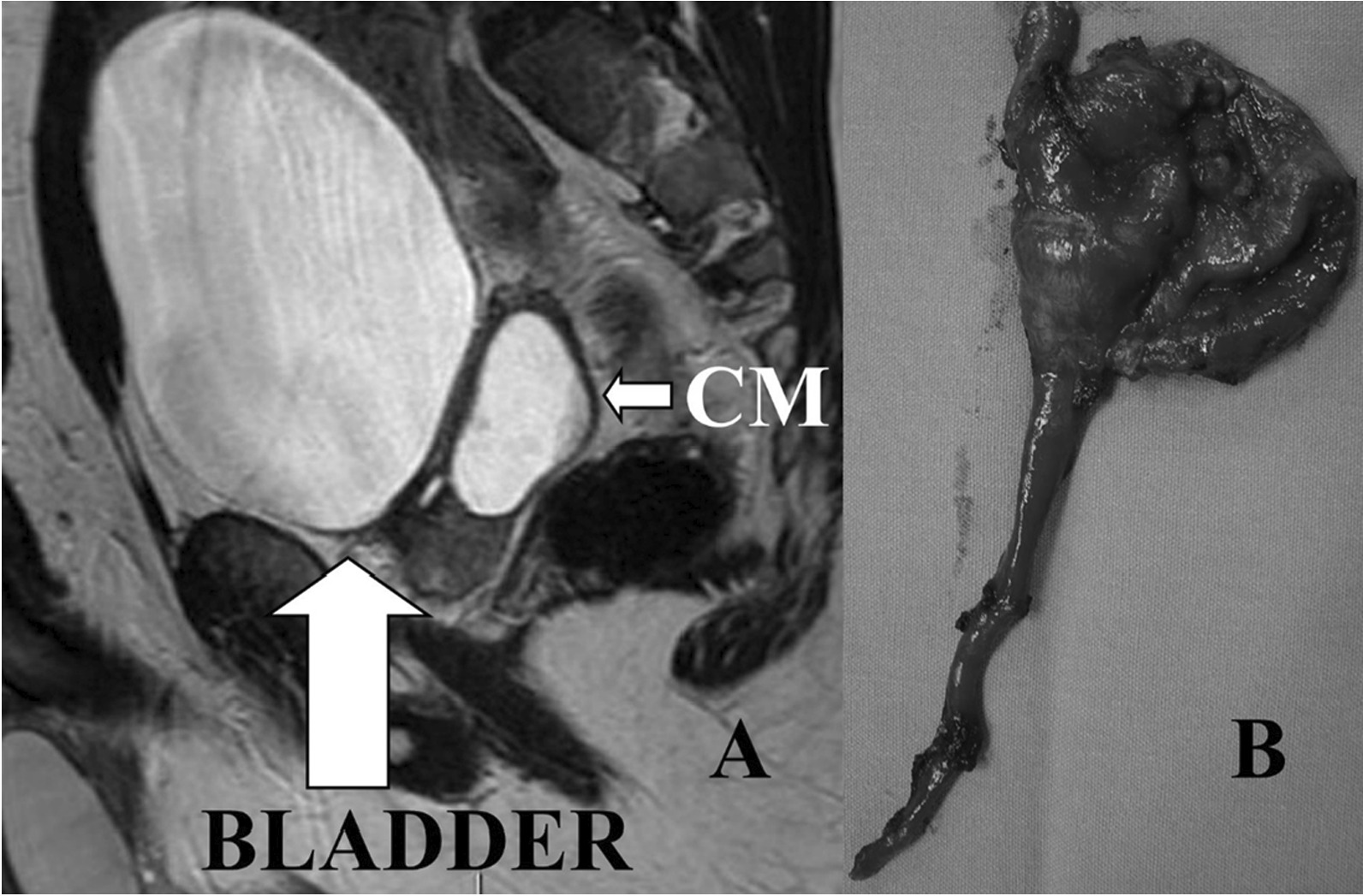
**A** MRI image of the cystic mass (CM). **B** Cystic mass originating from the deferent duct

A 20-year-old male presented to the urology unit in May 2012 with hypogastric pain, LUTS, ejaculatory disorders (reduced ejaculate volume and painful ejaculation), hematuria and persistent infections of the lower urinary tract. In his past history the patient underwent, three months after birth, a resection of the descending colon for Hirschsprung disease and right nephrectomy for renal dysplasia with histopathological report of interstitial nephritis. In 2009 he underwent a pelvic magnetic resonance imaging (MRI) for pelvic pain. The imaging revealed a right seminal vesicle cyst. For the persistence of symptoms, he underwent trans urethral resection of ejaculatory ducts (TUR-ED) without obtaining any improvement of the symptoms. Instead, a subsequent prostatitis and epididymitis appeared. Clinical examination revealed normal external genitalia; at a digital rectal examination (DRE) a swollen periprostatic mass was noticed. Semen analysis and blood tests did not show anomalies. A second MRI showed a liquid bilobated mass near the right seminal vesicle (54 × 38 × 47 mm), increased in volume compared to previous one, with a small hypointense signal, delimitated by a not-enhanced wall, with a fistulous communication with the prostatic urethra (Fig. 1a). Transrectal ultrasound showed a normal prostate without a clear visualization of the left seminal vesicle. Adjacent to the right seminal vesicle, a large bilobated cystic mass containing dense fluid was confirmed. Urethrocystoscopy and retrograde urethrocystogram showed a communication between the cystic structure and the prostatic urethra. Taking into account the patient’s previous surgeries, after semen cryopreservation, we decided to perform an open retropubic exeresis of the cyst because of the persistence of clinical symptoms (Fig. 1b). Histopathology examination reported a benign cyst coated by a cylindrical epithelium with diffuse squamous metaplasia and modest chronic inflammation of the cystic wall compatible with deferent duct cyst. On 15th post-operative day, due to a pelvic purulent collection a surgical drainage was performed. A subsequent fistula of the prostatic urethra developed and was managed with 6 weeks of bladder catheterization. At the last clinical consult the patient did not report any sexual or ejaculatory disorder.

### Case 2

**Fig. 2:**
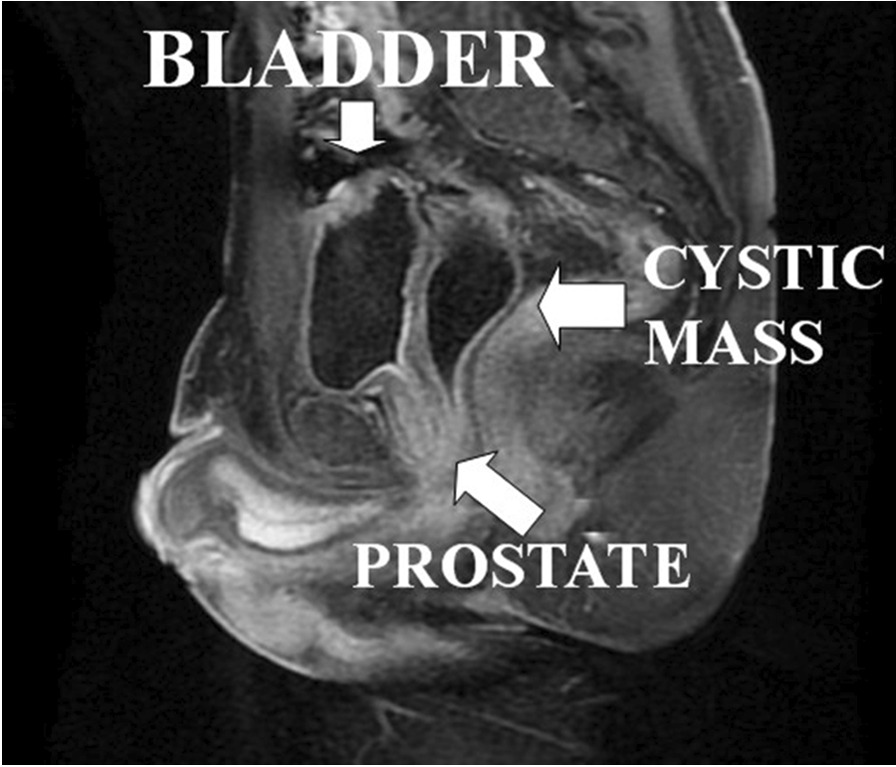
MRI shows the communication between the prostatic urethra and the cystic mass

In May 2013, a 28-year-old male presented to our operative unit with a history of urinary tract infections and reduced ejaculatory volume. He underwent urethroplasty for coronal hypospadias in childhood and right orchiectomy for testicular atrophy after orchidopexy failure in adolescence. Abdominal ultrasonography revealed a cystic mass posterior to the bladder. After computed tomography (CT) that failed to show abnormal findings, a pelvic MRI was performed, confirming a right paramedian retrovesical cystic mass of 30 × 80 mm, delimitated by a thin wall and suspicious for a Mullerian remnant (Fig. 2). Urethrocystoscopy showed a cavity on the right side of the verumontanum, and an open end 5 Ch ureteral catheter was inserted. Contrast injection through the uretheral catheter permitted to highlight a pseudocystic mass, with regular profile in communication with the prostatic urethra. A sperm cryopreservation was performed before surgery. The patient underwent a laparoscopic excision of the cystic mass and the pathological examination revealed a benign simple cyst of the right seminal vesicle with aspects of transitional epithelium and presence of pseudo-prostatic gland tissue. Post-operative recovery was regular and bladder catheter was removed 3 weeks after the surgery. The patient was asymptomatic and without urinary tract infections one year after the surgery.

## Discussion and conclusions

In both genders the genital tract originates from the mesonephric duct (Wolff duct) and the paramesonephric duct (Mullerian duct), which undergo a specific differentiation trajectory. Both systems are connected to the urogenital sinus and to the external genitalia, contributing to their development.

The Vas deferens, seminal vesicles and ejaculatory ducts derive from the mesonephric duct which opens on the side of the prostatic utricle. The epididymal appendix, bladder hemitrigone and ureteral buds also derive from this structure. For this reason, an anomaly in the mesonephric duct’s evolution may induce both internal genital malformations and renal agenesis.

Anomalies of these interactions cause malformations and alterations of sexual differentiation.

Among these complex syndromes, Zinner syndrome is a rare condition which shows some similarities with the first case described and is characterized by the following triad: obstruction of ejaculatory ducts, seminal vesicles cysts and omolateral renal agenesis [3].

In patients affected by Zinner syndrome the first symptoms appear more often from the 2nd to the 4th decade of life, at the beginning of their sexual activity [3]. Symptoms include abdominal, pelvic and perineal pain, painful ejaculation, reduced sperm volume and LUTS. The first patient described presented a clinical condition that could be a variant of Zinner syndrome, considering the presence of deferens duct cyst, seminal vesicle agenesis and omolateral renal dysplasia. Another anamnestic peculiarity of this patient is the presence of Hirschprung disease (congenital megacolon). In the literature there are studies which hypothesize an association between urinary tract malformations and congenital megacolon. This is related with the presence of a correlation between a gene deletion of RET (rearranged during transfection) or GDNF (Glial Derived Neurotrophic Factor) and alterations of renal organogenesis and the enteric innervation [4, 5]. More recently, associations between urinary tract malformations, congenital megacolon and 16p11.2 microdeletions have been discovered [6]. Moreover, the fact that seminal vesicles cysts are associated with ipsilateral renal agenesis in 68% of cases should be taken into account; in the absence of genitourinary abnormalities, this condition should be considered as an acquired disease [7].

Suprapubic or transrectal ultrasound is often the initial diagnostic imaging tool. Urethrocystoscopy and voiding cystourethrography are also useful diagnostic tests for these conditions. Nevertheless, the gold standard for the diagnosis of male pelvic floor cysts is MRI [1, 2]. Differential diagnosis between intraprostatic and extraprostatic cysts is performed with clinical and radiological findings, while histopathological characterization of the cystic wall is usually not significant. However, few cases of malignant cyst of Muller duct (adenocarcinomas and squamous cells carcinomas) have been described. Surgical treatment is recommended in symptomatic patients and in case of obstructive azoospermia. There are different treatment modalities including: trans-rectal drainage, transurethral resection of ejaculatory ducts (TUR-ED), robotic, laparoscopic and open surgery. Endoscopic treatment could be reserved to small lesions. This approach is characterized by lower morbidity, but it has the disadvantage of a higher recurrence rate [7], as reported in our first case.

In our opinion, open surgery is recommended in large masses and in patients who underwent previous surgery where a laparoscopic approach could be more challenging and have a higher complication rate. The following surgical approaches have been described: extravesical, transvesical, trans-rectal, perineal. The urethral fistula and the late purulent pelvic collection described in our case could be related to a local and systemic infection confirmed by the clinical, laboratory and intra-operative findings. The surgical drainage of the purulent collection, antibiotic therapy and prolonged bladder catheterization have permitted a complete clinical resolution. During the last years, robotic and laparoscopic surgery offered advantages such as image magnification, reduced blood loss, minor invasiveness and morbidity and better preservation of anatomical structures. Nowadays, laparoscopic treatment is the gold standard while robot-assisted surgery is limited to few cases and is associated to higher costs [8, 9].

Surgical treatment is reserved for symptomatic patients. In asymptomatic patients surveillance is an acceptable option. Pre-operative counseling is important in order to inform the patient about the potential complications such as infertility and erectile dysfunction. Patients must be adequately informed about risks and benefits of the surgical approach in order to reach a shared diagnostic and therapeutic decision making. In this context semen cryopreservation is useful and advisable before surgery.

## Data Availability

Data sharing is not applicable to this article as no datasets were generated or analyzed during the current study.

## Abbreviations

LUTS: Lower urinary tract symptoms
MRI: Magnetic resonance imaging
TUR-ED: Trans urethral resection of ejaculatory ducts
DRE: Digital rectal examination
CT: Computed tomography
SRY: Sex determining region of Y
TDF: Testis-determining factor on the Y
AMH: Anti-mullerian hormone
RET: Rearranged during transfection
GDNF: Glial derived neurotrophic factor
